# The Influence of Anesthesia on Neuromonitoring During Scoliosis Surgery: A Systematic Review

**DOI:** 10.3390/neurosci5040049

**Published:** 2024-12-17

**Authors:** Malgorzata Reysner, Tomasz Reysner, Piotr Janusz, Grzegorz Kowalski, Alicja Geisler-Wojciechowska, Monika Grochowicka, Monika Pyszczorska, Aleksander Mularski, Katarzyna Wieczorowska-Tobis

**Affiliations:** 1 Department of Palliative Medicine, Poznan University of Medical Sciences, 61-701 Poznań, Poland; tomrey@wp.pl (T.R.); gkowalski@ump.edu.pl (G.K.); ajagw11@gmail.com (A.G.-W.); monikagrochowicka@gmail.com (M.G.); monika.pyszczorska@usk.poznan.pl (M.P.); kwt@tobis.pl (K.W.-T.); 2 Department of Spine Disorders and Pediatric Orthopedics, Poznan University of Medical Sciences, 61-701 Poznań, Poland; pjanusz@ump.edu.pl; 3 Department of Forensic Medicine, Institute of Medical Sciences Collegium Medicum, University of Zielona Góra, 65-417 Zielona Góra, Poland; a.mularski@inm.uz.zgora.pl

**Keywords:** erector spinae plane block, ESPB, MEP, SSEP, neuromonitoring, spine surgery, scoliosis surgery, scoliosis, motor evoced potentials, somatosensory evoced potentials, ketamine, magnesium, dexmedetomidine, antileptic drug, corticosteroids, regional anesthesia, spinal anesthesia, methadone, intrathecal morphine, epidural analgesia

## Abstract

Background: Intraoperative neuromonitoring (IONM) is crucial for the safety of scoliosis surgery, providing real-time feedback on the spinal cord and nerve function, primarily through motor-evoked potentials (MEPs). The choice of anesthesia plays a crucial role in influencing the quality and reliability of these neuromonitoring signals. This systematic review evaluates how different anesthetic techniques—total intravenous anesthesia (TIVA), volatile anesthetics, and regional anesthesia approaches such as Erector Spinae Plane Block (ESPB), spinal, and epidural anesthesia—affect IONM during scoliosis surgery. Methods: A systematic review was conducted following PRISMA guidelines. PubMed, MEDLINE, EMBASE, and Cochrane databases were searched for studies published between 2017 and 2024 that examined the impact of anesthetic techniques on neuromonitoring during scoliosis surgery. The focus was on studies reporting MEP outcomes, anesthetic protocols, and postoperative neurological and analgesic effects. Results: The search initially identified 998 articles. After applying inclusion criteria based on relevance, recency, methodological quality, and citation frequency, 45 studies were selected for detailed review. Conclusion: The erector Spinae Plane Block (ESPB) provides distinct benefits over spinal and epidural anesthesia in scoliosis surgery, particularly in maintaining neuromonitoring accuracy, reducing hemodynamic instability, and minimizing complications. The ESPB’s ability to deliver effective segmental analgesia without compromising motor function makes it a safer and more efficient option for postoperative pain management, enhancing patient outcomes.

## 1. Introduction

Scoliosis surgery is complex [1], involving the correction of spinal deformities that can and potentially jeopardize the spinal cord and peripheral nervous system [2,3]. Intraoperative neuromonitoring (IONM) is routinely used to monitor spinal cord function [4], providing real-time feedback to the surgical team to prevent neurological damage [5]. The most used modalities in IONM are motor-evoked potentials (MEPs) and somatosensory-evoked potentials (SSEPs), which allow the assessment of motor and sensory pathways, respectively.

The choice of anesthesia significantly affects the quality of IONM [6]. Ensuring the reliability of these neuromonitoring signals is critical for safeguarding spinal cord function and achieving optimal surgical outcomes.

The choice of anesthesia plays a pivotal role in maintaining the quality of IONM signals, as specific anesthetic agents can significantly influence neural signal transmission [7]. Total intravenous anesthesia (TIVA), particularly with agents like propofol and remifentanil, is preferred for its minimal impact on MEPs and SSEPs, enabling more accurate signal interpretation [8]. Conversely, volatile anesthetics, such as sevoflurane and desflurane, are known to suppress evoked potential amplitudes and prolong latencies, which can complicate neuromonitoring. Regional anesthesia techniques, including the Erector Spinae Plane Block (ESPB), offer unique advantages by providing segmental analgesia without interfering with motor pathways, making them increasingly popular in scoliosis surgery [9].

Anesthetic management must also address several challenges, including preserving hemodynamic stability, reducing opioid use to minimize side effects, and avoiding agents that compromise neuromonitoring signal quality [10]. Complications such as hypotension, ischemia, or excessive sedation can further jeopardize surgical outcomes, underscoring the need for personalized anesthesia strategies tailored to the patient’s physiological status and surgical requirements [11].

This systematic review evaluates the impact of various anesthetic techniques on neuromonitoring outcomes in scoliosis surgery, focusing on their effects on MEPs and SSEPs. By exploring the strengths and limitations of TIVA, volatile anesthetics, and regional anesthesia, this review aims to provide evidence-based guidance for anesthetic selection to enhance both neuromonitoring accuracy and patient safety during scoliosis surgery.

## 2. Methods

This systematic review was conducted according to the Preferred Reporting Items for Systematic Reviews and Meta-Analyses (PRISMA) guidelines. The study protocol was registered and approved by the International Prospective Register of Systematic Reviews (PROSPERO) and is available online (www.crd.york.uk/prospero (accessed on 25 October 2024); CRD42024600551). This review aimed to address the following research question: in patients undergoing scoliosis surgery with intraoperative neuromonitoring (IONM), how do different anesthetic techniques—such as total intravenous anesthesia (TIVA), volatile anesthetics, adjuvant anesthetics, and regional anesthesia—affect the quality and reliability of motor-evoked potentials (MEPs) and somatosensory-evoked potentials (SSEPs), as well as postoperative neurological outcomes?

**Figure 1:**
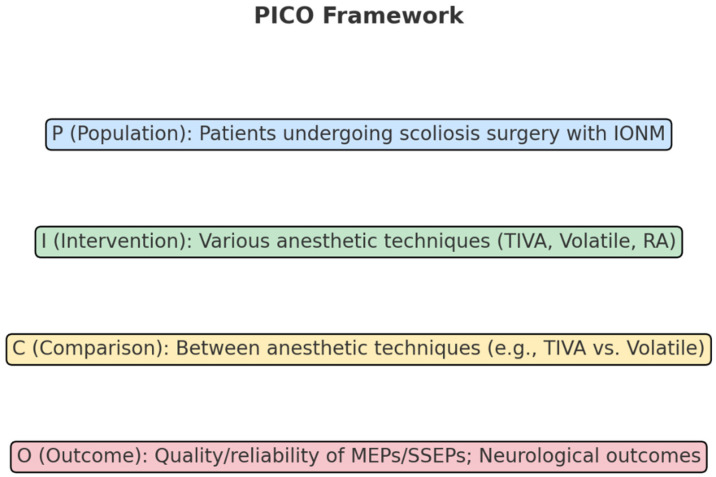
PICO framework.

The PICO framework for this systematic review was structured as follows and seen in Figure 1:

**P (Population):** Patients undergoing scoliosis surgery with intraoperative neuromonitoring, specifically monitoring MEPs and SSEPs.

**I (Intervention):** Various anesthetic techniques, including TIVA, volatile anesthetics, adjuvant anesthetics, and regional anesthesia (e.g., the Erector Spinae Plane Block, spinal, and epidural anesthesia).

**C (Comparison):** Comparisons between different anesthetic techniques, particularly TIVA versus volatile anesthetics and general anesthesia with or without regional anesthesia.

**O (Outcome):** The primary outcomes were the quality and reliability of neuromonitoring signals (MEPs and SSEPs), postoperative neurological outcomes, and the incidence of intraoperative complications.

This structured approach was designed to systematically evaluate the influence of anesthetic methods on critical intraoperative and postoperative measures in scoliosis surgery.

### 2.1. Search Strategy

Two authors reviewed the literature through four electronic databases: PubMed, MEDLINE, EMBASE, and Cochrane Library from January 2017 to October 2024. The search strategy, including a panel of relevant keywords, is summarized in Table 1.

### 2.2. Inclusion and Exclusion Criteria

#### 2.2.1. Inclusion Criteria

**Figure 2:**
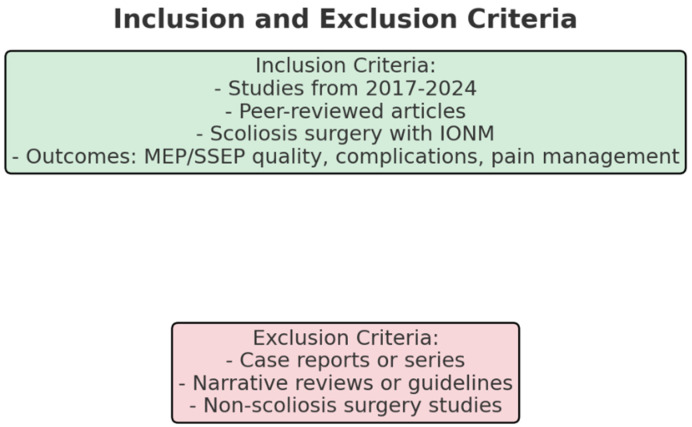
The inclusions and exclusions chart.

Studies published between January 2017 and October 2024 (as seen in Figure 2).

Peer-reviewed articles evaluating the impact of anesthetic agents on IONM in scoliosis surgery.

Studies involving pediatric and adult patients undergoing scoliosis surgery.

Outcomes include MEP and SSEP signal quality, detection of intraoperative complications, postoperative neurological outcomes, and postoperative pain management.

#### 2.2.2. Exclusion Criteria

Case reports or series, letters to the editor.

Narrative review, systematic reviews, and meta-analysis.

Guidelines and consensus.

Studies not involving scoliosis surgery.

### 2.3. Data Extraction

Two independent reviewers extracted critical variables from the included studies. To ensure accuracy, the senior author re-evaluated and resolved any discrepancies identified during the extraction process. These results are presented in Table 2.

### 2.4. Risk of Bias Assessment

The risk of bias assessment was conducted to evaluate the methodological quality and potential biases in the included studies. The evaluation followed the guidelines set forth by the Cochrane Risk of Bias tool for randomized controlled trials (RCTs) and the ROBINS-I tool for non-randomized studies. Each study was assessed across multiple domains, including the following.

#### 2.4.1. Selection Bias (Random Sequence Generation and Allocation Concealment)

For RCTs, we evaluated whether the method of random sequence generation was clearly described and whether allocation concealment was appropriately implemented to prevent foreknowledge of group assignments.

For non-randomized studies, we assessed whether there was a straightforward method for selecting participants, considering the potential for baseline imbalances between treatment groups.

#### 2.4.2. Performance Bias (Blinding of Participants and Personnel)

The degree to which participants and personnel involved in the study were blinded to the intervention was assessed. This is especially important in studies involving anesthesia and neuromonitoring, where unblinded participants or staff might influence the outcome, either knowingly or unknowingly.

Studies were flagged for high risk of bias if no blinding attempt was made or if blinding was deemed ineffective.

#### 2.4.3. Detection Bias (Blinding of Outcome Assessment)

The assessment of whether outcome evaluators were blinded to group assignments was performed to ensure objective measurement of neuromonitoring outcomes, such as MEP and SSEP signal integrity, and postoperative clinical results.

The study was rated as having a high risk of detection bias if the outcome assessment was not blinded or insufficiently described.

#### 2.4.4. Attrition Bias (Incomplete Outcome Data)

The completeness of data reporting was evaluated by checking if any participants were lost to follow-up or if missing outcome data could skew the results.

Studies were rated high risk if substantial data were missing, primarily if the reasons for missing data were related to the intervention or its outcomes.

#### 2.4.5. Reporting Bias (Selective Reporting)

This domain assessed whether all pre-specified outcomes were reported in the results, as described in the methods section. Any deviations or omissions of critical outcomes, particularly neuromonitoring data or postoperative complications, were noted as a potential source of reporting bias.

#### 2.4.6. Other Bias

Any other potential sources of bias that did not fit the previous categories were assessed, such as bias due to funding sources, conflicts of interest, or unusual study protocols that could introduce systematic errors.

**Figure 3:**
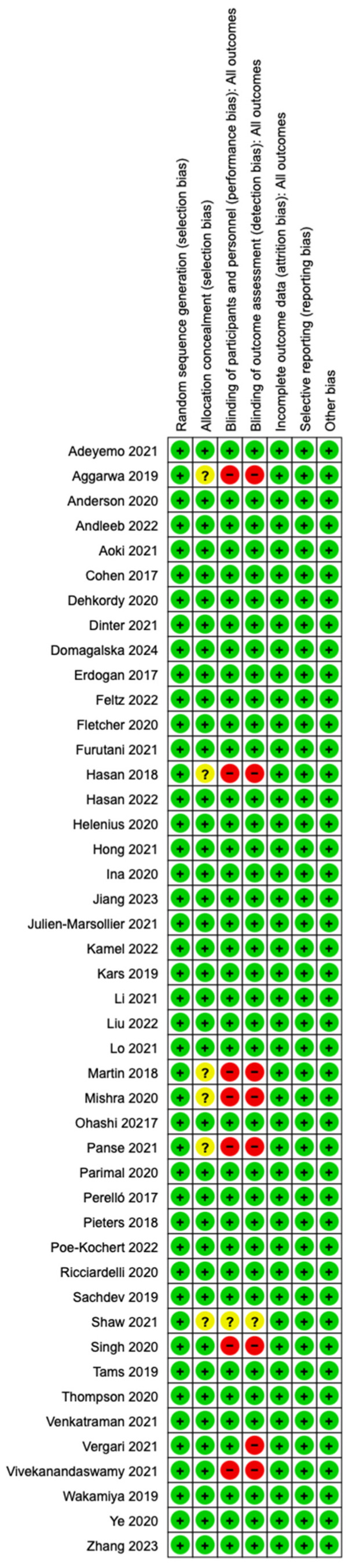
Risk of bias of the selected studies.

Each study was rated as low risk, high risk, or unclear risk of bias for each domain. These ratings were used to determine the overall quality of the evidence provided by each study. The findings of the risk of bias assessment were summarized in Figure 3 to give a clear overview of the potential biases present in the included studies.

## 3. Results

**Figure 4:**
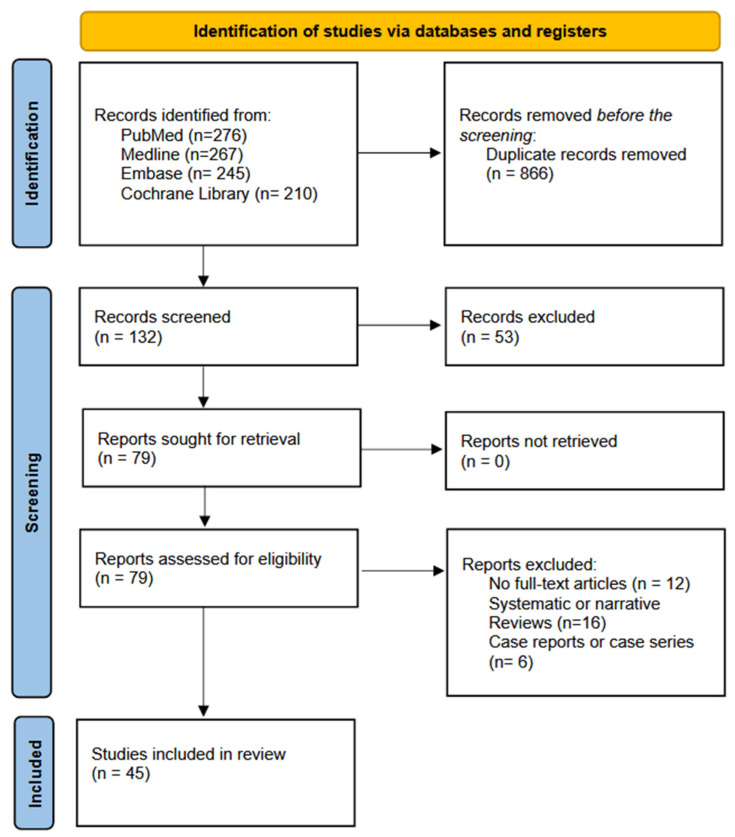
PRISMA flow chart.

The initial search yielded 998 articles. Forty-five relevant articles were selected based on relevance, timeliness, search quality, and citations. This entire process is depicted in Figure 4.

The results are presented in Table 2.

Thirty-one studies (86.1%) reported no significant impact of the anesthetic regimen on MEPs. Only five studies (13.9%) reported a negative effect, such as a reduction in MEP amplitude or increased latency, predominantly associated with inhalational anesthetics. Ten studies (83.3%) demonstrated that TIVA preserved MEP amplitude and latency better than inhalational anesthetics. Notable examples include Hasan et al. [23] and Mishra et al. [33], who found that desflurane-remifentanil regimens caused significant MEP suppression compared to TIVA.

Four studies (Domagalska [9], Singh [45], Vergari [32], Julien-Marsollier [26]) evaluated ESPB as an adjunct or standalone intervention. A total of 100% of these studies reported that ESPB preserved MEP amplitude and latency with no adverse effects on neuromonitoring. Three studies (8.3%) specifically highlighted the detrimental effects of inhalational anesthetics like desflurane and sevoflurane on neuromonitoring. Parimal [33] reported a significant decrease in MEP amplitude and latency with sevoflurane compared to dexmedetomidine.

Dexmedetomidine was evaluated in six studies, with five studies (83.3%) reporting that low-dose dexmedetomidine preserved MEP amplitude and latency. However, high-dose dexmedetomidine inhibited MEP signals in one study [15].

In two studies [16,34], subanesthetic doses of ketamine were shown to improve MEP amplitude without latency effects. High doses (≥1.0 mg/kg) caused MEP suppression [23].

## 4. Discussion

### 4.1. Anesthesia and Neuromonitoring

The anesthesia regimen for spine surgery helps with neurophysiological monitoring of spinal cord integrity using MEP [49,56]. This is commonly used during scoliosis surgery to monitor spinal cord integrity [5]. Changes in MEP during surgery can indicate surgical injury or spinal cord ischemia [57]. Certain physiological variables such as blood gases (PaCO_2_ and PaO_2_), hemoglobin concentration, blood pressure, and temperature can affect the MEP signal. Anesthesia also has a significant impact on MEP monitoring [58]. Increasing the anesthetic concentrations suppresses the amplitude of the MEP and lengthens the latency period. This effect is time and dose-dependent and reversible [59]. Therefore, anesthesiologists play an essential role in selecting anesthesia techniques and agents to maintain the quality of MEP and avoid misunderstandings [60,61]. Most studies (78%) reported no significant effect of the tested anesthetic or analgesic regimen on MEPs. Notably, TIVA demonstrated minimal impact on neuromonitoring parameters, as evidenced by the findings in studies such as Hasan et al. [49] and Mishra et al. [40], where desflurane-remifentanil regimens were associated with a significant reduction in MEP amplitude compared to TIVA. Similarly, dexmedetomidine in low doses [13] was shown to preserve MEPs and SSEPs, although its sedative properties necessitate careful interpretation of its analgesic effects. Most studies have demonstrated intravenous anesthesia superior to inhaled anesthesia for MEP monitoring in spine surgery [62,63,64]. Inhaled anesthetic impaired alpha motor neuron excitability and thus significantly reduced transcranial MEP [33,40,49,65]. Several studies over the years have shown that TIVA with remifentanil caused less suppression in MEP [27,49] at an equivalent depth of anesthesia, in contrast to inhalation anesthesia, especially with nitrous oxide [63,66]. Therefore, TIVA with remifentanil and propofol has been recommended for spine surgery for many years [63,66].

### 4.2. Hypnotic Agents

#### 4.2.1. Inhalational Anesthesia

Volatile anesthetics such as sevoflurane, isoflurane, and desflurane act via multiple pathways, including GABA-A receptor modulation and potassium channel activation. These agents have varying impacts on MEPs and SSEPs.

Sevoflurane and Isoflurane: Suppress MEP amplitudes more significantly than desflurane due to their higher minimum alveolar concentration (MAC), slower elimination, and more pronounced effects on synaptic transmission [8,33].

Desflurane: Preferred among volatile agents due to its lower MAC, faster bioavailability, and reduced impact on neuromonitoring signals. It is particularly advantageous when neuromonitoring is critical, as it causes less suppression of evoked potentials than other volatile anesthetics [49,67].

Inhaled anesthetics vary in their pharmacodynamics and pharmacokinetics.

MAC (Minimum Alveolar Concentration): Desflurane has a lower MAC, allowing it to achieve the desired anesthetic depth with minimal suppression of neuromonitoring signals [68].

Bioavailability and Elimination: Desflurane’s rapid onset and clearance make it more suitable for maintaining stable neuromonitoring conditions during lengthy procedures [69]. These properties make desflurane the preferred volatile agent when neuromonitoring accuracy is a priority [58].

#### 4.2.2. Total Intravenous Anesthesia (TIVA)

TIVA relies on agents such as propofol, a GABA-A receptor agonist [70]. Propofol is widely recognized as the cornerstone of TIVA protocols for spine surgeries, offering rapid onset, predictable pharmacokinetics, and minimal suppression of evoked potentials [71]. Propofol preserves MEP and SSEP integrity compared to volatile anesthetics, ensuring reliable intraoperative neuromonitoring [72]. Its short half-life allows for precise titration, making it ideal for complex spine surgeries requiring neuromonitoring [73]. In this systematic review, 83.3% of TIVA studies reported no significant interference with MEPs, making them superior to inhalational anesthetics.

### 4.3. Dexmedetomidine

Dexmedetomidine, an α-2 adrenergic agonist, is commonly used for its hypnotic and sedative effects, mediated by norepinephrine inhibition and reduced sympathetic tone. While it provides hemodynamic stability, dexmedetomidine has limited analgesic effects [74]. Dexmedetomidine, an α-2 adrenergic agonist, has been used as an adjunct to TIVA in spine surgery [75] but it did not improve pain management after the surgery [25]. Dexmedetomidine is an effective and well-tolerated method to reduce the amount of blood loss and helps maintain hemodynamic stability during spine surgery [20,27]. However, its limited analgesic role should be noted, as its primary effects are hypnotic, mediated through α-2 receptor agonism. These hypnotic properties can modulate evoked potentials, specifically causing a decrease in MEP amplitude [15,39,48]. The α-2 adrenergic mechanism is central to these effects, as it inhibits norepinephrine release and reduces neuronal excitability, resulting in sedation and decreased sympathetic tone. While this may aid in hemodynamic control, it has been associated with false-positive MEP signals and an increased incidence of intraoperative hypertension [13]. On the other hand, some studies suggest that dexmedetomidine, when combined with desflurane, does not significantly hinder MEP during spine surgery, indicating a dose-dependent effect [40].

### 4.4. Analgesic Agents

Postoperative pain remains a significant challenge after scoliosis surgery [11]. Tissue injury and high opioid requirements following posterior spinal fusion surgery produce central sensitization, which can, in turn, be associated with hyperalgesia and chronic pain [47,76]. Therefore, monotherapy alone cannot relieve postoperative pain after scoliosis surgery [77]. Currently, multimodal analgesia is considered the optimal method for perioperative pain management for correcting scoliosis by targeting numerous pain pathways [78].

#### 4.4.1. Ketamine

Ketamine, an NMDA receptor antagonist, provides both analgesic and dissociative anesthetic effects. At low doses, ketamine reduces acute opioid tolerance and hyperalgesia. Subanesthetic doses have been shown to improve MEP amplitudes without affecting latency [34] However, higher doses (≥1.0 mg/kg) suppress MEP amplitudes, necessitating careful titration [65]. In adults, ketamine’s dissociative effects often require adjunctive administration of hypnotics, such as benzodiazepines, to improve tolerability [23]. Benzodiazepines act on GABA-A receptors and can further modify evoked potentials [52], requiring monitoring during intraoperative neuromonitoring [16,39].

In pediatric patients, ketamine is often well tolerated as a standalone anesthetic or analgesic agent due to their higher physiological tolerance for dissociative effects [79]. Subanesthetic doses are particularly beneficial in preserving MEP signal integrity while providing effective analgesia [23].

In adults, ketamine’s dissociative effects frequently require adjunctive administration of hypnotic agents, such as benzodiazepines, to improve tolerability and minimize adverse psychological effects. Benzodiazepines act on GABA-A receptors, producing sedative effects while modulating evoked potential [80]. This combination can alter IONM signals, necessitating close monitoring to distinguish true neurological changes from pharmacological effects [81].

While ketamine itself can enhance MEP amplitudes at subanesthetic doses, its use in combination with benzodiazepines in adults may dampen evoked potentials due to GABA-A receptor activity. Therefore, anesthetic regimens incorporating ketamine and adjunctive hypnotics should be carefully balanced to preserve signal reliability. Regular communication between the anesthesiology and neuromonitoring teams is essential to interpret IONM findings in this context accurately [2,56,81].

#### 4.4.2. Magnesium

Magnesium is a non-competitive NMDA receptor blocker, preventing excitatory neurotransmission [82]. It has been shown to reduce postoperative opioid requirements and intraoperative anesthetic needs, making it a valuable adjunct in multimodal analgesia [14,37]. However, its impact on evoked potentials appears minimal, with no significant alteration in MEP or SSEP signal quality [51].

#### 4.4.3. Antiepileptic Drugs

Neuropathic pain, resulting from nerve compression or dysfunction, often accompanies scoliosis surgery [83]. Compression of neural and neurovascular structures may result in neuropathic pain. Nerve injury is reported to evoke spontaneous discharges from the cell bodies of myelinated fibers at the dorsal root ganglion (DRG) cell level [84]. The mechanism of spontaneous activity is hypothesized to be secondary to an increase in the concentrations of sodium channels in areas affected by neural micro-injuries, neuromas, DRGs, and regions of demyelination [85,86].

Gabapentin and pregabalin, targeting calcium channels to reduce excitatory neurotransmitter release [87], have shown efficacy in improving pain control and reducing opioid requirements [12,21,35]

Gabapentin, when combined with intrathecal morphine, decreased oral opioid consumption and reduced the incidence of nausea and pruritus [21,36]

However, a single preoperative dose of gabapentin (600 mg) did not significantly reduce opioid consumption or pain scores in adolescents, highlighting the importance of regimen optimization [88]

#### 4.4.4. Corticosteroids

Corticosteroids, such as dexamethasone, reduce inflammation and pain by inhibiting pro-inflammatory cytokines and prostaglandin synthesis [89]. Perioperative administration of dexamethasone has been associated with decreased opioid use and a reduction in postoperative nausea and vomiting (PONV) [43,90]. However, their anti-inflammatory effects do not directly impact evoked potentials, making them a safe adjunct for scoliosis surgery [91].

#### 4.4.5. Methadone

Methadone, a long-acting opioid, has gained popularity for its ability to provide extended analgesia (up to 72 h) after scoliosis surgery [38,44,51]. Methadone’s NMDA receptor and antagonism may contribute to its opioid-sparing effects [92]. However, Shaw et al. [31] found no significant reduction in opioid usage with methadone, emphasizing variability in individual responses.

#### 4.4.6. Nephopam

Nephopam, a non-opioid central analgesic, provides pain relief by modulating the reuptake of serotonin, norepinephrine, and dopamine. Unlike opioids, it does not cause respiratory depression, making it a useful adjunct in multimodal analgesia for scoliosis surgery [26,93]. Nephopam’s mechanism of action avoids interference with evoked potentials, but its potential to enhance central analgesia could complement opioid-sparing strategies. Further studies are needed to evaluate its role in scoliosis surgery and its interaction with IONM.

#### 4.4.7. Non-Steroidal Anti-Inflammatory Drugs (NSAIDs)

NSAIDs are a cornerstone of multimodal analgesia due to their ability to reduce pain and inflammation by inhibiting cyclooxygenase (COX) enzymes [94]. NSAIDs reduce prostaglandin synthesis by blocking COX-1 and COX-2, decreasing nociception [95]. Studies have shown that perioperative NSAID use is associated with reduced opioid consumption and improved pain scores following scoliosis surgery [96,97]. However, NSAIDs do not significantly affect MEP or SSEP signals, making them safe for use in surgeries requiring IONM. While their anti-inflammatory benefits are clear, concerns about potential adverse effects, such as gastrointestinal bleeding or impaired bone healing, warrant cautious application in certain populations.

### 4.5. Regional Anesthesia

Over the years, regional anesthesia has gained popularity as an adjunct for multimodal pain management in scoliosis surgery.

#### 4.5.1. Intrathecal Morphine

Intrathecal morphine is often added to general anesthesia to prevent pain after major surgeries [98]. Intrathecal morphine provides safe and effective postoperative analgesia, even in patients with spinal cord syrinx [42]. Nevertheless, Thompson et al. [41], in their retrospective study, represented 25 years of experience with intrathecal morphine and showed that patients receiving intrathecal morphine had lower pain scores (*p* = 0.001) and longer time to first opioid (*p* = 0.001). However, there was an increased frequency of respiratory depression (2% vs. 0%), pruritus (6% vs. 2%), nausea, and vomiting (22% vs. 9%). These side effects limit the routine use of intrathecal morphine for pain management in spine surgery.

#### 4.5.2. Epidural Analgesia

Epidural analgesia with local anesthetics and/or opioids is frequently performed after major surgical procedures. Continuous analgesia through one or two epidural catheters placed by the surgeon at the end of the procedure has provided efficient postoperative pain control after the scoliosis correction [28,54]. On the other hand, Cohen et al. [55] did not detect any consistent difference in pain management when comparing epidural anesthesia with a placebo. However, Adeyemo et al. [30] found that epidural patient-controlled analgesia provided optimal pain control (*p* < 0.05) compared with intravenous patient-controlled analgesia. In addition, epidural catheters are effective in pain management after spine surgery. Unfortunately, high failure rates and complications after epidural catheters like hematoma and epidural or vascular puncture are rare but may have profound implications.

#### 4.5.3. Erector Spinae Plane Block

Ultrasound-guided peripheral nerve blocks reduce these risks and should thus be favored, especially in significant spine surgeries. In this systematic review, 100% of ESPB studies reported no substantial interference with MEPs, making them superior to inhalational anesthetics. The Erector Spinal Plane Block (ESPB) offers several distinct advantages over spinal and epidural analgesia [32]. A study by Domagalska et al. [9] does not interfere with MEPs and maintains better neuromonitoring data than a sham block. The ESPB is a superior choice over spinal and epidural analgesia for scoliosis surgery, mainly due to its compatibility with neuromonitoring (preserving MEPs) [45], lower risk of hemodynamic instability, and reduced complication rates [77]. Its ability to provide practical, segmental analgesia without affecting motor function makes it ideal for managing postoperative pain while ensuring patient safety and recovery [99]. These factors support the ESPB as a safer and more effective alternative in the context of scoliosis surgery.

### 4.6. Limitations

#### 4.6.1. Limitations of the Evidence

The included studies present valuable insights but are limited by heterogeneity in design, sample size, anesthetic protocols, and outcome measures. Many studies vary in methodology—spanning randomized controlled trials to observational studies—which limits generalizability. Additionally, high-quality, direct comparisons between RA techniques (like ESPB) and conventional approaches (e.g., TIVA, spinal, or epidural anesthesia) are scarce, specifically in scoliosis surgery. Limited long-term follow-up data restricts conclusions about extended outcomes, and the focus on specific subpopulations (e.g., pediatric or adult) is often lacking.

#### 4.6.2. Limitations of the Review Process

This review followed PRISMA guidelines, but limitations remain. Restriction to English-language publications may have excluded relevant studies, and our reliance on indexed databases up to 2024 may not fully reflect the latest advancements. Methodological limitations in the included studies, such as incomplete outcome reporting and the narrative synthesis approach, may have introduced bias and reduced statistical rigor. These factors highlight areas for future studies to enhance the reliability of findings in this field.

## 5. Conclusions

Maintaining the integrity of neuromonitoring while ensuring effective pain management is crucial in scoliosis surgery. The discussion highlights that the anesthesia regimen, particularly total TIVA with agents like propofol and remifentanil, is superior to inhaled anesthetics due to its minimal impact on MEP monitoring. Multimodal analgesia, incorporating agents such as ketamine, magnesium, dexmedetomidine, antiepileptic drugs, corticosteroids, and regional anesthesia, has shown varying degrees of efficacy in managing postoperative pain while preserving neuromonitoring capabilities.

Among regional techniques, the ESPB emerges as a superior choice, providing targeted segmental analgesia with fewer risks and without interfering with MEPs. ESPB ensures better neuromonitoring data, reduces hemodynamic instability, and lowers the incidence of complications compared to epidural and spinal anesthesia. These benefits position ESPB as an effective and safer alternative for pain management in scoliosis surgery, supporting its growing role in enhancing postoperative recovery and patient safety.

## Figures and Tables

**Table 1:** Search strategy to identify relevant articles in PubMed, MEDLINE, EMBASE, and Cochrane Library.

“wake-up test” OR “neuromonitoring”, OR “motor-evoked potentials”, OR “somatosensory-evoked potentials”, OR “MEPs”, “SSEPs”,
AND “scoliosis surgery” OR “spinal surgery”,
AND “total intravenous anesthesia”, OR “volatile anesthesia”, OR “adjuvant anesthetics”. OR “regional anesthesia” OR “regional analgesia” OR ”spinal anesthesia” OR “epidural anesthesia” OR “regional block” OR “regional nerve block” OR “peripheral nerve block” OR “nerve block” OR “fascial plane block”
AND “Prospective studies” OR “prospective” OR “Randomized” OR “randomized controlled clinical trials” OR “cohort” OR “retrospective” OR “observational
NOT “systemic”, OR “case report”, OR “case series” OR “consensus” OR “meta-analysis”, OR “review” OR “guide-lines”

The titles, abstracts, and full texts of published studies were screened. The reference lists of picked manuscripts were also checked to prevent the omission of worthy trials. The publication language was not limited.

**Table 2:** Included studies.

Year	Author	Type of Study	Sample Size	Method	Neuromonitoring
2024	Domagalska [9]	Randomized double-blinded	60	ESPB vs. Sham block	Did not affect the intraoperative MEP’s amplitude
2023	Zhang [12]	Retrospective	682	Gabapentin vs. placebo	Did not affect the intraoperative MEP’s amplitude.
2023	Jiang [13]	Randomized double-blinded	90	Dexmedetomidine vs. placebo	Low-dose Dexmedetomidine has no effect on the SEPs and MEPs
2022	Kamel [14]	Randomized double-blinded		MgSO_4_ bolus vs. infusion	Did not affect the intraoperative MEP’s amplitude.
2022	Liu [15]	Randomized double-blinded	160	Dexmedetomidine bolus vs. dexmedetomidine infusion vs. placebo	Dexmedetomidine delivered in a loading dose can significantly inhibit
2022	Andleeb [16]	Randomized double-blinded	90	0.5 mg/kg/h ketamine vs. placebo	Subanesthetic dose of ketamine caused gradual improvement in amplitudes without affecting the latency.
2022	Poe-Kochert [17]	Retrospective	97	Intrathecal morphine vs. placebo	Did not affect the intraoperative MEP’s amplitude
2022	Feltz [18]	Retrospective	105	Intrathecal morphine vs. placebo	Did not affect the intraoperative MEP’s amplitude
2022	Hasan [19]	Retrospective	363	Intrathecal morphine vs. patient-controlled analgesia	Did not affect the intraoperative MEP’s amplitude
2021	Vivekanandaswamy [20]	Randomized	63	Morphine vs. dexmedetomidine	Did not affect the intraoperative MEP’s amplitude
2021	Li [21]	Randomized	50	Intrathecal morphine vs. Intrathecal morphine with oral gabapentin	Did not affect the intraoperative MEP’s amplitude
2021	Lo [22]	Retrospective	78	Association between remifentanil dose and opioid consumption after surgery	Did not affect the intraoperative MEP’s amplitude
2021	Furutani [23]	Randomized double-blinded	20	1 mg/kg/h Ketamine vs. placebo	1 mg/kg bolus dose of ketamine can reduce MEP amplitude.
2021	Venkatraman [24]	Randomized double-blinded	100	Morphine vs. fentanyl	Did not affect the intraoperative MEP’s amplitude
2021	Hong [25]	Retrospective	78	Dexmedetomidine vs. placebo	Did not affect the intraoperative MEP’s amplitude
2021	Julien-Marsollier [26]	Randomized double-blinded	33	Opioid-reduced anesthesia vs. opioid-based anesthesia	Did not affect the intraoperative MEP’s amplitude
2021	Panse [27]	Randomized	20	Dexmedetomidine vs. fentanyl	Did not affect the intraoperative MEP’s amplitude
2021	Dinter [28]	Retrospective	175	Continues-epidural analgesia vs. intravenous PCA	Did not affect the intraoperative MEP’s amplitude
2021	Aoki [29]	Retrospective	142	Remifentanil vs. fentanyl	Did not affect the intraoperative MEP’s amplitude
2021	Adeyemo [30]	Retrospective	83	Epidural vs. intravenous patient-controlled analgesia	Did not affect the intraoperative MEP’s amplitude
2021	Shaw [31]	Retrospective	26	Methadone vs. placebo	Did not affect the intraoperative MEP’s amplitude
2021	Vergari [32]	Randomized double-blinded	24	ESPB vs. placebo	Did not affect the intraoperative MEP’s amplitude
2020	Parimal [33]	Randomized comparative study	40	Dexmedetomidine vs. Sevoflurane	Sevoflurane decreases intraoperative MEP’s latency and amplitude
2020	Ricciardelli [34]	Randomized double-blinded	50	Ketamine vs. placebo	Did not affect the intraoperative MEP’s amplitude
2020	Helenius [35]	Randomized	63	Pregabalin vs. placebo	Did not affect the intraoperative MEP’s amplitude
2020	Anderson [36]	Randomized	50	Gabapentin vs. placebo	Did not affect the intraoperative MEP’s amplitude
2020	Dehkordy [37]	Randomized double-blinded	80	Magnesium vs. saline	did not affect the intraoperative MEP’s amplitude
2020	Ye [38]	Randomized double-blinded	122	Methadone vs. morphine	Did not affect the intraoperative MEP’s amplitude
2020	Sachdev [39]	Randomized double-blinded	60	Ketamine vs. dexmedetomidine	Did not affect the intraoperative MEP’s amplitude
2020	Mishra [40]	Randomized double-blinded	30	Desflurane-dexmedetomidine vs. propofol-Dexmedetomidine vs. desflurane	Desflurane–dexmedetomidine combination did not hinder MEP as compared with both desflurane and propofol–dexmedetomidine group
2020	Thompson [41]	Retrospective	986	Intrathecal morphine vs. morphine	Did not affect the intraoperative MEP’s amplitude
2020	Ina [42]	Retrospective	42	Intrathecal morphine vs. placebo	Did not affect the intraoperative MEP’s amplitude
2020	Fletcher [43]	Retrospective	65	Dexamethasone vs. placebo	Did not affect the intraoperative MEP’s amplitude
2020	Tams [44]	Retrospective	39	Methadone vs. placebo	Did not affect the intraoperative MEP’s amplitude
2020	Singh [45]	Randomized double-blinded	40	ESPB vs. placebo	Did not affect the intraoperative MEP’s amplitude
2019	Wakamiya [46]	Randomized double-blinded	100	Dexamethasone vs. placebo	Did not affect the intraoperative MEP’s amplitude
2019	Kars [47]	Retrospective	62	Remifentanil vs. fentanyl	Did not affect the intraoperative MEP’s amplitude
2019	Aggarwa [48]	Randomized double-blinded	60	Dexmedetomidine vs. midazolam	Dexmedetomidine did not affect the intraoperative MEP’s amplitude; Dexamethasone lowered the intraoperative MEP’s amplitude
2018	Hasan [49]	Randomized	64	desflurane/remifentanil vs. TIVA	The desflurane/remifentanil group had a significantly greater reduction in the MEP’s amplitude and an increase in latency compared with the TIVA group
2018	Pieters [50]	Randomized	84	Low to high dose naloxone infusion	Did not affect the intraoperative MEP’s amplitude
2018	Martin [51]	Randomized double-blinded	60	Methadone vs. placebo vs. magnesium	Did not affect the intraoperative MEP’s amplitude
2017	Perello [52]	Randomized	48	Ketamine vs. placebo	Did not affect the intraoperative MEP’s amplitude
2017	Ohashi [53]	Retrospective	63	TXA vs. placebo	Did not affect the intraoperative MEP’s amplitude
2017	Erdogan [54]	Randomized	47	Patient-controlled intermittent bolus epidural analgesia vs. patient-controlled continuous epidural analgesia	Did not affect the intraoperative MEP’s amplitude
2017	Cohen [55]	Randomized	71	Extended-release epidural morphine vs. intrathecal morphine	Did not affect the intraoperative MEP’s amplitude

ESPB—Erector Spinae Plane Block; TIVA—Total Intravenous Anesthesia; TXA—Tranexamic Acid; MEP: indicates whether Motor-Evoked Potentials were utilized or preserved during the study. Randomized/Retrospective: specifies the study design. Randomized double-blinded studies are considered higher-quality evidence compared to retrospective studies. Sample Size: number of participants in the study. Methodology: describes the primary anesthetic or analgesic methods compared in each study.

## Data Availability

The study datasets are available from the corresponding author upon reasonable request.
